# Supplementary material for: Recognition of a disulfiram ethanol reaction in the emergency department is not always straightforward
Source: PLoS One. 2020 Dec 3;15(12):e0243222. doi: 10.1371/journal.pone.0243222 (PMC7714420; doi:10.1371/journal.pone.0243222)
Supplement: Attachment — Submitted filename: Response to Reviewers.docx [file pone.0243222.s002.docx]

| contact | telefoon | e-mail | |
| --- | --- | --- | --- |
| Tania Desmet  Emergency Department | +32 (0)9 332 15 59 | Tania.desmet@uzgent.be | |
| ons kenmerk | fax | datum | pagina |
| PONE-D-20-14245 | +32 (0)9 332 49 80 | 12 oktober 2020 | 1/3 |
| betreft  Response to Reviewers | | | |

Emily Chenette

Deputy Editor-in-Chief

PLOS ONE

Sector

Kritieke diensten

Dienst

Spoedgevallen

Diensthoofd

Prof. dr. P. De Paepe

Stafleden

Prof. dr. S. Hachimi Idrissi

Prof. dr. P. Van de Voorde

Prof. dr. W. Buylaert

Dr. C. Lyphout

Dr. T. Desmet

RECEPTIE

+32 (0)9 332 50 24

Dear Editor-in-chief,

Dear Reviewers,

First of all, we would like to thank you and the reviewers for the time spent on reviewing the manuscript and the thoughtful comments helping us improving the article. In the attached letter we would like to answer the comments and questions listed.

**Reviewer #1**

1. Page 13, line 222: Disulfiram is also increasingly used for the following conditions: cocaine addiction, tumor diseases and pathological gambling. Please cite papers for each disease. This makes the examination all the more relevant.

***Other conditions in which disulfiram is used for (including references) have been added to	the manuscript (line 229-231).***

2. Page 15, line 283: There are biochemical markers, which can improve and verify a hidden DSR, please cite this paper and discuss it: Urinary Ethylglucuronide Assessment in Patients Treated With Disulfiram: A Tool to Improve Verification of Abstention and Safety, Mutschler et al, Clin Neuropharmacol . Nov-Dec 2010;33(6):285-7. doi: 10.1097/WNF.0b013e3181fc9362.

***The authors thank you for this contribution and have adapted the text accordingly (line 313-	316).***

3. Diskussion: I think it would be also interesting to short discuss this paper by Mutschler et al: Experienced Acetaldehyde Reaction Does Not Improve Treatment Response in Outpatients Treated With Supervised Disulfiram, Clin Neuropharmacol . Jul-Aug 2011;34(4):161-5. doi: 10.1097/WNF.0b013e3182216fd5.

***The authors thank you for this addition and have incorporated this interesting observation in the manuscript	(line 234-235 ; reference 47).***

4. It would be interesting to discuss the effect of the drug in more detail (biological effect, psychological effect). Furthermore pharmacogenetic aspects.

***The authors agree with these comments. The manuscript has been adapted based on the suggestions provided.***

***Although this is a very interesting comment, we feel that a discussion on the biological and psychological effects	is beyond the scope of this paper. We would therefore suggest not including it. It is not clear to us to which specific	pharmacogenetic aspects of disulfiram the reviewer is alluding.***

**Reviewer #2**

1. Describing the DER as an ‘aversive reaction’ tout court reinforces the widespread but incorrect notion that disulfiram (DSF) works by ‘aversion’ – i.e. the repeated coupling of drinking with an unpleasant response. In reality, most patients never deliberately test out DSF’s acataldehydaemic potential because the vicarious knowledge of that potential deters them from drinking while ALDH inhibition persists. The mechanism is deterrence, not aversion.

***The authors agree with these comments. The manuscript has been adapted and the term « aversive reaction »	has been omitted throughout the entire manuscript.***

2. DSF and its active metabolite S-methyl N,Ndiethylthiolcarbamate sulfoxide do not have long half-lives and are usually undetectable after 48 hours. However, because it is an irreversible ‘suicide inhibitor’ of ALDH, ALDH inhibition persists until the body produces new ALDH in adequate quantities. This process is probably genetically determined and can be as short as a couple of days or as long as 10 days – occasionally more.

***The authors thank you for this contribution and have adapted the text accordingly (line 62-66 & line 308-311).***

3. Carbon disulphide – another metabolite of DSF – may be easier to detect than DSF itself. It, too, persists for about two days after the last dose.

***The authors thank you for this contribution and have adapted the text accordingly (line 352-353).***

4. DSF dosage information was not systematically collected in the study but one patient is recorded as having 400mg daily. Although bioavailability can vary with the manufacturing process, a starting dose of 200-250-mg is usually appropriate, though some supervised DSF programmes use 400/400/5-600mg on Monday/Wednesday/Friday. Dosage will only need increasing if the patient risks drinking and gets no reaction or only a very mild one.

***The dosage of disulfiram was not systematically reported in this study. This fell beyond the scope of the study.	The authors agree that a gradual increase of the dosage of disulfiram may be preferable.***

5. Although I have never had occasion to use fomepizole, I think it deserves a slightly longer discussion, including its mode of action. I also think there may be a case for using it in any severe case of suspected DER pending further investigations or confirmation.

***The authors agree with these comments. The manuscript has been adapted based on the suggestions provided	(line 328-329).***

6. The authors say that even small quantities of alcohol can cause a DER. ‘Small’ is a rather elastic but in reality, small amounts such as in sauces never cause a reaction, or only an extremely mild one, though patients who inadvertently swallow small amounts of an alcoholic sauce sometimes have a panic attack that can superficially resemble a DER. Some very recent papers have drawn attention to the fact that inadvertently inhaling alcohol in hand-sanitisers can cause a DER, probably because of a rapid but transient build-up of acetaldehyde in lung tissues that then passes directly and quickly to the heart and brain. While COVID persists, this may be worth mentioning. However, the total amount of alcohol inhaled – compared with the amount in even one glass of wine – is so small that severe DERs from this route seem very unlikely and alcohol absorption directly through the skin is negligible.

***The authors thank you for this addition and have incorporated this interesting observation in the manuscript	(line 66-70).***

Kindest regards,

Dr. Tania Desmet
