# Supplementary material for: Recognition of a disulfiram ethanol reaction in the emergency department is not always straightforward
Source: PLoS One. 2020 Dec 3;15(12):e0243222. doi: 10.1371/journal.pone.0243222 (PMC7714420; doi:10.1371/journal.pone.0243222)
Supplement: Attachment — Submitted filename: Response to Reviewers.docx [file pone.0243222.s003.docx]

| contact | telefoon | e-mail | |
| --- | --- | --- | --- |
| Tania Desmet  Emergency Department | +32 (0)9 332 15 59 | Tania.desmet@uzgent.be | |
| ons kenmerk | fax | datum | pagina |
| PONE-D-20-14245R1 | +32 (0)9 332 49 80 | 2 november 2020 | 1/1 |
| betreft  Response to Reviewers | | | |

Emily Chenette

Deputy Editor-in-Chief

PLOS ONE

Sector

Kritieke diensten

Dienst

Spoedgevallen

Diensthoofd

Prof. dr. P. De Paepe

Stafleden

Prof. dr. S. Hachimi Idrissi

Prof. dr. P. Van de Voorde

Prof. dr. W. Buylaert

Dr. C. Lyphout

Dr. T. Desmet

RECEPTIE

+32 (0)9 332 50 24

Dear Editor-in-chief,

Dear Reviewers,

First of all, we would like to thank you and the reviewers for the time spent on reviewing the manuscript and the thoughtful comments helping us improving the article. In the attached letter we would like to answer the comments and questions listed.

**Reviewer #2**

1. I am sorry if I seem difficult to satisfy but despite your agreement that the term 'aversive' is inappropriate, it still appears in Line 67, Please replace it with 'unpleasant'.

***The authors thank you for this contribution and have adapted the text accordingly (line 67).***

2. Similarly, you have again claimed a long half-life for disulfiram, despite accepting that it is the effect on ALDH that is prolonged, not the half-life. Please correct this.

***The authors thank you for this contribution and have adapted the text accordingly (line 258-	259).***

Kindest regards,

Dr. Tania Desmet
